# The regulation of inhibitor of apoptosis proteins (IAPs) during the apoptosis of *Cotesia chilonis*

**DOI:** 10.3389/fphys.2023.1328167

**Published:** 2023-12-19

**Authors:** Ming-Xing Lu, Fu-Jing He, Feng Zhu, Yu-Zhou Du

**Affiliations:** ^1^ College of Plant Protection and Institute of Applied Entomology, Yangzhou University, Yangzhou, China; ^3^ Plant Protection and Quarantine Station of Jiangsu Province, Nanjing, China; ^4^ Joint International Research Laboratory of Agriculture and Agri-Product Safety, Yangzhou University, Yangzhou, China; ^2^ Wuxi Vocational Institute of Commerce, Wuxi, China

**Keywords:** inhibitor of apoptosis protein, Cotesia chilonis, thermal stress, developmental stages, biological stress, RNA interference

## Abstract

Inhibitor of apoptosis proteins (IAPs) are crucial components of apoptosis that perform vital roles in the regulation of caspase activity in organisms. In this study, two IAPs genes were identified from *Cotesia chilonis*, the dominant parasitic wasp of *Chilo suppressalis*. *Cc*IAP1 gene is a typical IAP and contains two BIR domains and a RING domain, whereas *Cc*IAP gene is an atypical IAP1 only containing two BIR domains. Phylogenetic analysis indicated that *Cc*IAP1 and *Cc*IAP were grouped with other Hymenopteran IAPs and IAP1 in *C. suppressalis.* Real-time quantitative PCR revealed that *Cc*IAP1 and *Cc*IAP genes were both highly induced at −6°C and 30°C, and expression was highest at the third instar stage. The expression of *Cc*IAP1 and *Cc*IAP genes were significantly induced during parasitism of *C. suppressalis*, and the 7-d time point resulted in the highest expression levels for both genes, in which was an advanced stage of larval development of *C. chilonis*. RNAi experiments showed that *Cc*IAP1 gene was the key IAP in the regulation of apoptosis of *C. chilonis* and its host. In conclusion, *Cc*IAP1 and *Cc*IAP correlate with the development of *C. chilonis* and their responses to temperature stress.

## 1 Introduction

Apoptosis is a basic biological process necessary for the normal development of organisms and the maintenance of the internal environment of tissues (Vaux and Korsmeyer, 1999). This term was first proposed by Kerr, Wyllie and Currie in 1972, and this unique mode of cell death is triggered by internal and external environmental changes and can remove senescent, redundant and damaged cells under the regulation of genes (Kerr et al., 1972). As an important arthropod immune response, apoptosis is an active and orderly process of cell death, which can prevent the proliferation of virus and is regulated by a series of proteins. Among them, the inhibitor of apoptosis protein family (IAPs) is critical in the regulation of apoptosis in arthropods (Everett and McFadden, 1999; Vandergaast et al., 2015). Furthermore, previous studies have shown that apoptosis was a generally regulated cell death program, involving the dynamic balance between the activities of pro- and anti-apoptotic factors (Hamajima et al., 2016).

Inhibitor of apoptosis proteins (IAPs) are the crucial inhibitors in the programmed cell death cascade and are named for their anti-apoptotic activity (Danial and Korsmeyer, 2004). The earliest IAP gene was identified when they screened P^35^ homologous genes from baculovirus in 1993 (Crook et al., 1993). Up to now, six types of IAP genes have been identified in baculoviruses: IAP1, IAP2, IAP3, IAP4, IAP5, and the newly described IAP6 (Luque et al., 2001; Ikeda et al., 2013; Clem, 2015; Chen et al., 2020). In addition, subsequent studies on other species show that IAPs not only exist in baculoviruses, but also homologs of IAPs have also been found in the other organisms such as fruit flies, nematodes, yeast, mammals and humans (Silke and Vaux, 2001; Zhen and Zhang, 2004). Among them, the relative research mainly focuses on fruit flies, silkworms, mosquitoes and ticks, but rare studies are found in insects (Hay et al., 1995; Huang et al., 2001; Li et al., 2007). Structurally, IAPs family is highly conversed, and there are usually one to three repeating sequences called baculoviral IAP repeat at the N-terminus, which are also called BIR (Baculovirus IAP Repeats) motifs or BIR domains. Additionally, the C-terminus of most IAPs also contains a structure that called RING domain (Williams et al., 2009).

*Chilo suppressalis* (Walker) is a significant rice pest which is widely distributed in the southern and northern regions of China and is severely damaged. The current prevention situation is still severe (Lu et al., 2013; Luo et al., 2014), which has triggered new biological control methodologies such as the use of natural enemies (He et al., 2013; Gao et al., 2019). *Cotesia chilonis* (Munakata) is widely distributed in China and has become the dominant parasitic wasp of *C. suppressalis* as global temperatures have risen (Chen et al., 2002; Pan et al., 2016; Pan et al., 2018). Therefore, the use of *C. chilonis* to prevent and control *C. suppressalis* has certain application value (Li et al., 2019; He et al., 2021). However, under the global climate warming, the mechanism of mutual adaptation between *C. suppressalis* and *C. chilonis* is not obscure against the background of global warming. And the influence of different factors on IAP genes expression of *C. chilonis* is well worth exploring. Thus, in this study 2 *C. chilonis* IAP genes (*Cc*IAP1 and *Cc*IAP) were found and identified and their structural characteristics were described. The expression of these two genes in response to temperature stress along with their expression in different developmental stages were studied. The impact of *C. chilonis*-mediated endoparasitism on the expression of these two genes in *C. suppressalis* was also examined. Moreover, RNAi was used to further clarify the function of *C. chilonis* IAP genes and explore relationships between pro- and anti-apoptotic genes in the host and parasitoid.

## 2 Materials and methods

### 2.1 Experimental insects

*C. suppressalis* and *C. chilonis* were collected from a suburb of Yangzhou (32.39°N, 119.42°E) and reared under the laboratory conditions at 27 ± 1°C, 60%–70% RH and a 16:8 h (light/dark) photoperiod (Pan et al., 2018). *C. suppressalis* were reared on an artificial diet (Gao et al., 2019). *C. chilonis* adults were supplied with a 10% honey/water solution and propagated using fifth instar larvae of *C. suppressalis* as hosts. Both *C. suppressalis* and *C. chilonis* were reared successively for three or more generations. Ethical review and approval was not required for the study on animals in accordance with the local legislation and institutional requirements.

### 2.2 Sample treatments

For different developmental stages treatment, a single fifth instar of *C. suppressalis* was placed in a test tube and *C. chilonis* adults were added in a 2:1 female/male ratio for breeding. Insects were incubated for 6 h at 27°C in darkness to facilitate parasitism of *C. suppressalis*; once parasitism occurred, *C. suppressalis* larvae were allowed to feed on artificial diet. Insects were maintained using the 27°C regimes described above until *C. chilonis* emerged from *C. suppressalis*. Three parasitized *C. suppressalis* were dissected from the two treatments on a daily basis, and the development of five randomly-selected *C. chilonis* individuals was inspected; different developmental stages of *C. chilonis* (first instar larvae, second instar larvae, third instar larvae, pupae and adults) were collected and stored at −80°C until needed. Treatments either contained 30 (larvae) or five individuals (adults and pupae). All treatments were replicated three times.

For parasitic time treatment, after parasitism for 1 h, 10 h, 24 h, 2 days, 3 days, 4 days, 5 days, 6 days, 7 days, 8 days and 9 days, the fifth instar larvae of *C. suppressalis* were collected. Four replicate groups for each interval of parasitism were collected and stored at −80°C.

For temperature treatment, 1-day old adults of *C. chilonis* were subjected to −13, −12, −9, −6, −3, 0, 27, 30, 33, or 36°C for 1 h in a constant-temperature incubator (Pan et al., 2018); samples were then placed in a climate-controlled incubator and allowed to recover at 27°C for 1 h. Each treatment contained 30 1-day old adults, and all treatments were replicated 3 times.

### 2.3 RNA isolation and synthesis of first strand cDNA

Total RNA was extracted from *C. chilonis* and *C. suppressalis* using the SV Total RNA Isolation System (Promega, USA). The integrity of RNA was verified by comparing ribosomal RNA bands in ethidium bromide-stained gels, and RNA purity was examined using spectrophotometric measurements at A_260_ and A_280_ nm (NanoDrop One, Thermo Fisher Scientific, United States). The first strand of cDNA was generated with an oligo (dT) _18_ primer (Fermentas, Canada).

### 2.4 Cloning and genome amplification

Partial gene sequences were obtained from the *C. chilonis* transcriptome, and specific primers were designed to verify fragments using the first strand of cDNA as template (Supplementary Table S1). Full-length cDNA sequences of genes were obtained with 5′- and 3′- RACE (SMARTer™ RACE, Clontech), and gene-specific primers were designed for verifying full-length cDNA sequences using the 5′-RACE template (Supplementary Table S1). Genomic DNA of *C. chilonis* adults was extracted using the Axyprep™ Multisource Genomic DNA Kit (Axygen, United States), and primers (Supplementary Table S1) were designed to amplify genomic fragments of *Cc*IAP1 and *Cc*IAP for subsequent cloning or sequencing.

### 2.5 Sequence analysis of genes

ORFs (Open reading frames) were identified with ORF Finder (https://www.ncbi.nlm.nih.gov/orffinder/), and deduced amino acid sequences were aligned with ClustalX. Sequence analysis tools on the ExPASy Molecular Biology Server including Translate, Compute pI/MW, and Blast (Swiss Institute of Bioinformatics), were used to analyze the predicted sequences. Motif Scan (https://prosite.expasy.org/) and InterPro (http://www.ebi.ac.uk/interpro/) were used to identify motifs characteristic of the IAPs family. Phylogenetic trees were constructed by the neighbor-joining minimum evolution, maximum likelihood and maximum parsimony methods with 1000 bootstrap replicates using MEGA 7.0 (Kumar et al., 2016).

### 2.6 Real-time qPCR analysis

Total RNA was isolated from the different treatments as described above, and the Bio-Rad iScript™ cDNA Synthesis Kit (Bio-Rad, USA) was used to reverse transcribe 0.5 µg total RNA into first strand cDNA. The primers used for real-time quantitative PCR (Supplementary Table S1) were designed based on the full-length cDNA sequence of genes. Real-time PCR reactions were conducted using SYBR Green I in a 20 μL volume that included 10 μL iTaq™ SYBR^®^ Green Supermix, 6 μL ddH_2_O, 2 μL cDNA template and 1 μL each of the corresponding forward and reverse primers. Reaction conditions for PCR were as follows: 3 min initial denaturation step at 95°C, followed by 40 cycles of 15 s denaturation at 95°C, and 30s annealing at the Tm for each gene (Supplementary Table S1). Melting curve analysis was carried out to evaluate the homogeneity of the amplified PCR products. Each PCR reaction was replicated in triplicate. GAPDH was regarded as the high-temperature reference gene and H3 was regarded as the remaining treatment reference gene (Li et al., 2019).

### 2.7 Synthesis of dsRNA and RNAi experiments

Two full-length *C. chilonis* IAP genes were analyzed by the online website (http://sidirect2.rnai.jp); the regions for RNA silencing were determined, and gene-specific primers with an integrated T7 promoter were designed (Supplementary Table S1). A dsRNA specific to *gfp* encoding green fluorescence protein served as a control (Supplementary Table S1). The amplified products were sequenced and used as template DNA for dsRNA synthesis as recommended for the MEGAscript^®^RNAi Kit (Thermo Fisher Scientific, USA). The quality and integrity of dsRNA molecules were evaluated by spectrophotometry and gel electrophoresis. The dsRNAs were stored at −80°C until needed.

Third instar larvae of *C. suppressalis* parasitized by *C. chilonis* for 9 days were selected for the RNAi experiments. The dosages of the dsRNA solution and nanomaterial (provided by Prof. Jie Shen, China Agricultural University) were 0.25 μL and 13.71 μL, respectively, for one parasitized third instar of *C. suppressalis*. The dsRNA solution and nanomaterial were mixed for 10–15 min, and detergent (provided Prof. Jie Shen) was added and incubated for 10–15 min; this solution was then placed on top of parasitized third instar larvae of *C. suppressalis* for 10–15 min in darkness. At 24 h post-treatment, parasitized *C. suppressalis* were dissected. *C. suppressalis* and *C. chilonis* individuals were collected for RNA extraction, and RNAi efficiency was analyzed by qRT-PCR. Photos were taken with a fluorescence microscope (Nikon ECLIPSE Ts2R) (Deerfield, IL, German) and a KEYENCE VHX-5000 system. The experiment was performed with three replicates per treatment. Non-parasitized *C. suppressalis* and dsGFP were used as controls.

### 2.8 Statistical analysis

Gene expression was normalized using the 2^−ΔΔCt^ method. Differences in mean values were analyzed with the independent-sample *t*-test and ANOVA. Levene’s test was used to evaluate homogeneity of variances, and Tukey’s test was used to assess significant differences. SPSS v.16.0 (SPSS, Chicago, IL, USA) was used for statistical analysis and presented as means ± SE (standard error).

## 3 Results

### 3.1 Characteristics of sequenced genes

**FIGURE 1:**
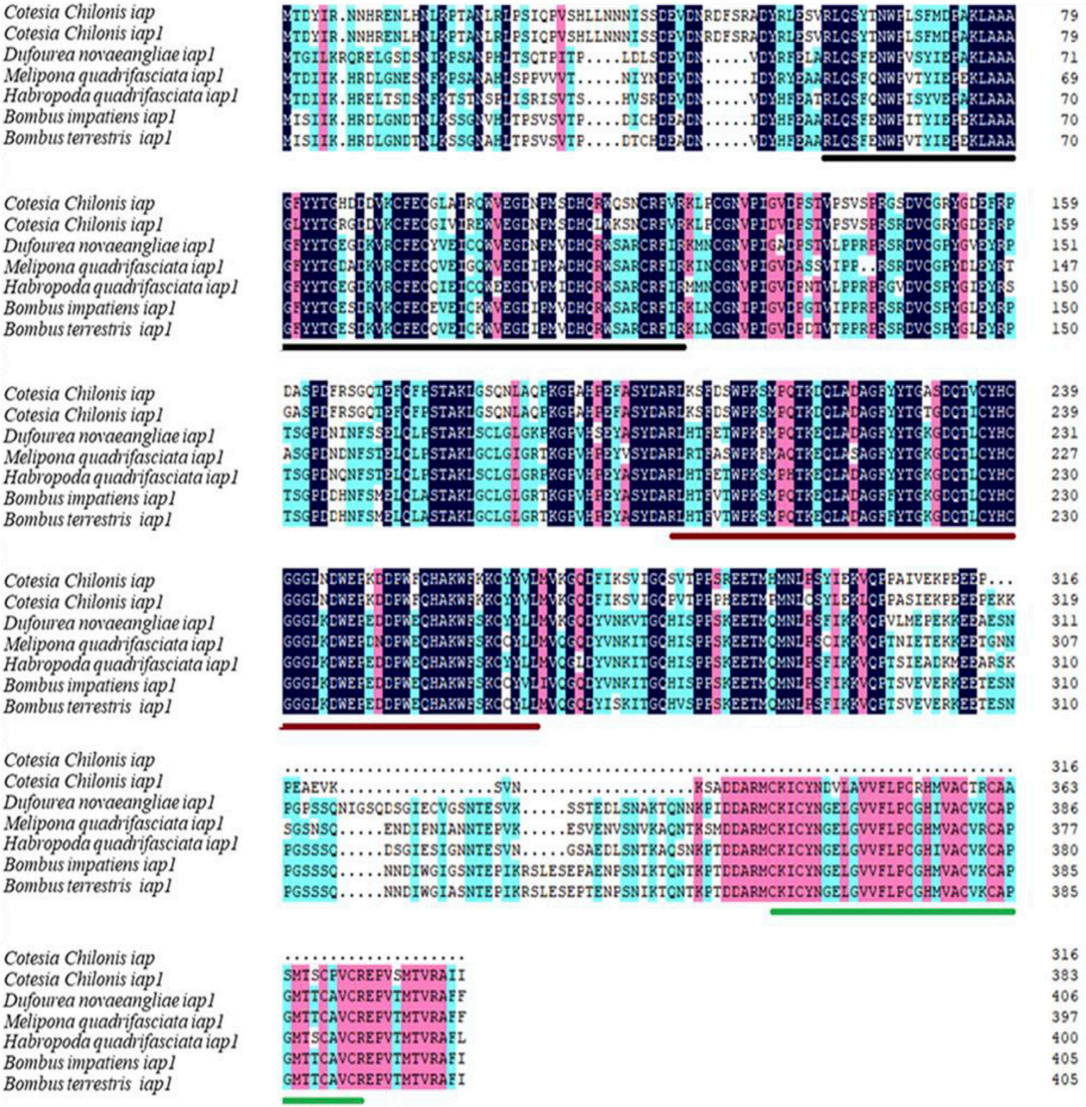
Amino acid sequence alignment of *Cc*IAP1 and *Cc*IAP from *Cotesia chilonis* with orthologous proteins in *Dufourea novaeangliae*, *Melipona quadrifasciata*, *Habropoda quadrifasciata*, *Bombus impatiens* and *Bombus terrestris*. Identical amino acids are shaded with the same color. The two BIR sequences are marked by black and red line, respectively. The RING domain is underscored in green, respectively. Accession numbers of species are noted in Supplementary Table S2.

The full-length cDNA sequence of *Cc*IAP1 gene was 1404 bp (GenBank accession no. MZ076685) and contained a 210-bp 5′ untranslated region (UTR), a 1155-bp ORF, and 39-bp 3′ UTR. The full-length cDNA of *Cc*IAP gene was 1184 bp (GenBank accession no. MZ076686) and contained a 190-bp 5′ UTR, a 951-bp ORF, and a 43-bp 3’ UTR. Comparison of cDNA and genomic sequences revealed the absence of introns in both *Cc*IAP1 and *Cc*IAP genes. The predicted *Cc*IAP1 protein contained 384 amino acids, with theoretical molecular mass of 43.32 kDa and an isoelectric point (pI) of 6.33. The deduced *Cc*IAP protein contained 316 amino acids with a predicted mass of 35.88 kDa and pI of 5.73. Analysis using Motif Scan and InterPro revealed that *Cc*IAP1 and *Cc*IAP both contained two BIR repeat signatures (residues 59–123 and 202–267 for *Cc*IAP1; residues 59–123 and 202–267 for *Cc*IAP). Furthermore, *Cc*IAP1 also contained a putative zinc finger (residues 337–372). Multiple sequence alignments revealed that *Cc*IAP1 shared 55.53%, 55.61%, 54.63%, 53.73% and 54.22% identity with IAP1 in *Dufourea novaeangliae*, *Melipona quadrifasciata*, *Habropoda quadrifasciata*, *Bombus impatiens* and *Bombus terrestris*, respectively. *Cc*IAP shared 46.39%, 44.88%, 45.61%, 43.86% and 44.34% identity with IAP1 in *Dufourea novaeangliae*, *Melipona quadrifasciata*, *Habropoda quadrifasciata*, *Bombus impatiens* and *B. terrestris*, respectively (Figure 1).

### 3.2 Phylogenetic analysis of genes

**FIGURE 2:**
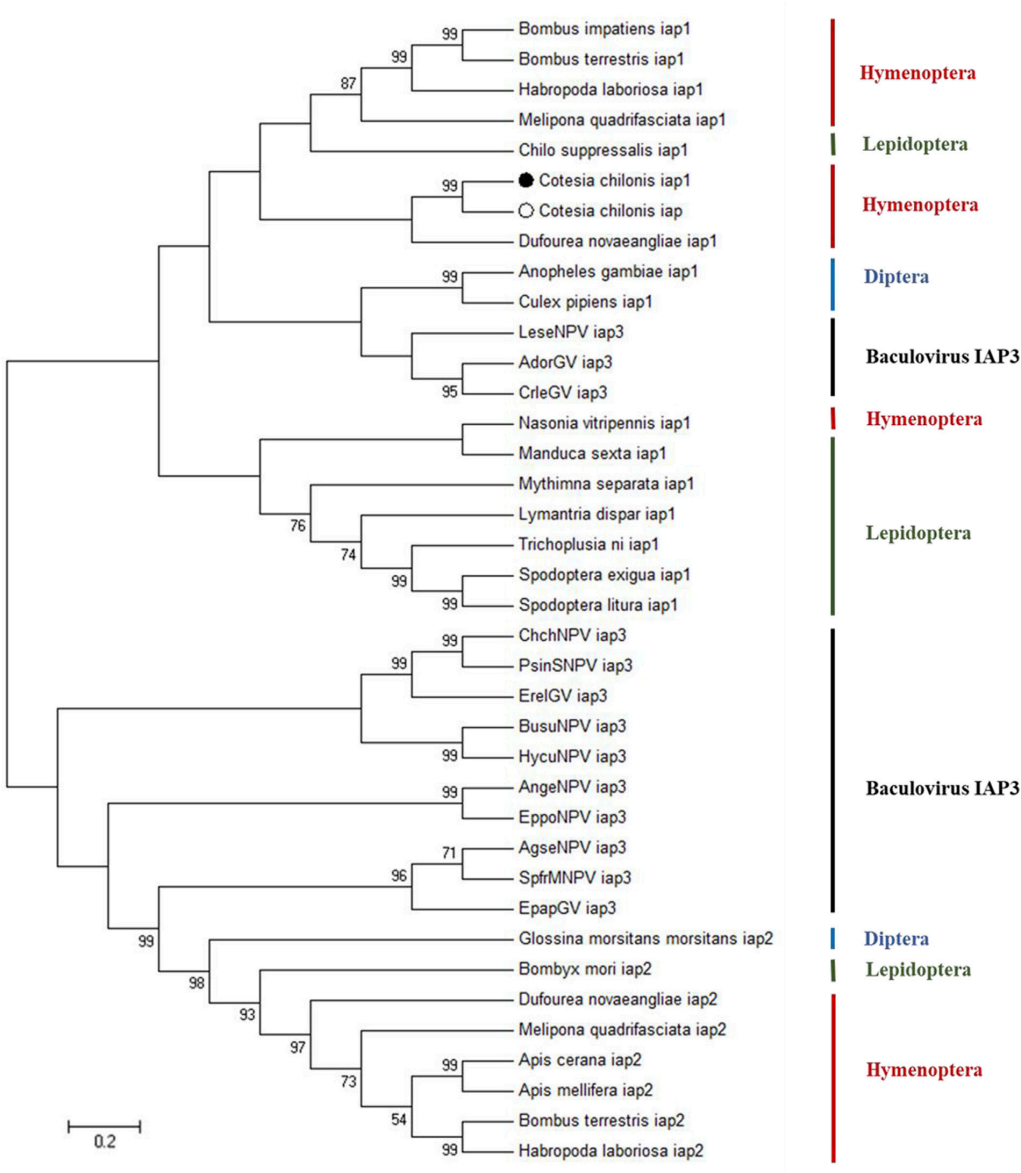
Phylogenetic tree based on the different species IAPs and baculovirus IAP3 proteins. Trees were generated with MEGA 7.0, and solid circles indicate the location of *Cc*IAP1 and *Cc*IAP Numbers on the branches are bootstrap values (1000 replicates), and only bootstrap values >50 are shown. Accession numbers are provided in Supplementary Table S2.

Previous research indicated that insect IAPs clustered with baculovirus IAP3, which suggests acquisition of IAPs via horizontal gene transfer (Chen et al., 2020). To explore evolutionary relationships, BlastP (https://blast.ncbi.nlm.nih.gov/Blast.cgi) was used to search for proteins related to baculovirus IAP3. Phylogenetic analysis of IAPs was performed using neighbor-joining, maximum likelihood, maximum parsimony and minimum evolution methods, and similar results were obtained with the different methods. The dendrogram in Figure 2 is representative of the phylogenetic groupings and was obtained with the neighbor-joining method; it clearly shows that *Cc*IAP1 and *Cc*IAP are closely related to IAP1s in other members of the Hymenoptera and IAP1 in *C. suppressalis*. Furthermore, phylogenetic analysis revealed that baculovirus IAP3s are closely related to insect IAPs in the orders Lepidoptera, Diptera and Hymenoptera. Analysis of the IAPs using ClustalX and MEGA 7.0 further confirmed that *Cc*IAP1 and *Cc*IAP share high similarity with IAP1s in other hymenopteran insects.

### 3.3 Gene expression during different developmental stages

**FIGURE 3:**
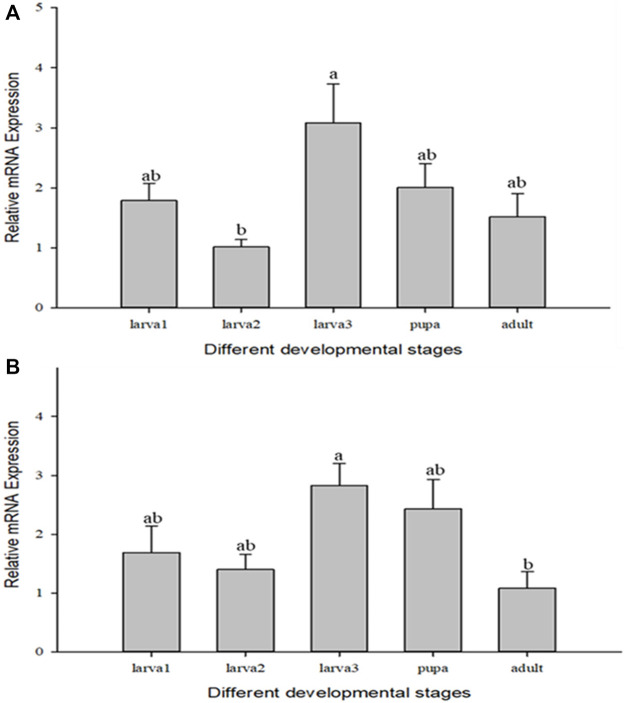
Relative mRNA expression levels of *Cc*IAP1 **(A)** and *Cc*IAP **(B)** at different developmental stages. Statistics are presented as means ± SE. Columns labeled with different letters indicate significance between temperatures using one-way ANOVA followed by Tukey’s multiple comparison analysis (*p* <0.05).

*Cc*IAP1 and *Cc*IAP genes both showed variable expression in the different developmental stages of *C. chilonis*. *Cc*IAP1 expression was significantly upregulated in the third instar larvae as compared to the second instar larvae (Figure 3A, *F*_4,11_ = 3.365, *p* = 0.05), and *Cc*IAP expression was significantly higher in the third instar larvae as compared to adults (Figure 3B, *F*_4,13_ = 3.891, *p* < 0.05). *Cc*IAP1 and *Cc*IAP genes expression levels in the third instar larvae were 3.03- and 2.62-fold higher than expression in the second instar larvae, respectively.

### 3.4 Gene expression in response to different parasitic time

**FIGURE 4:**
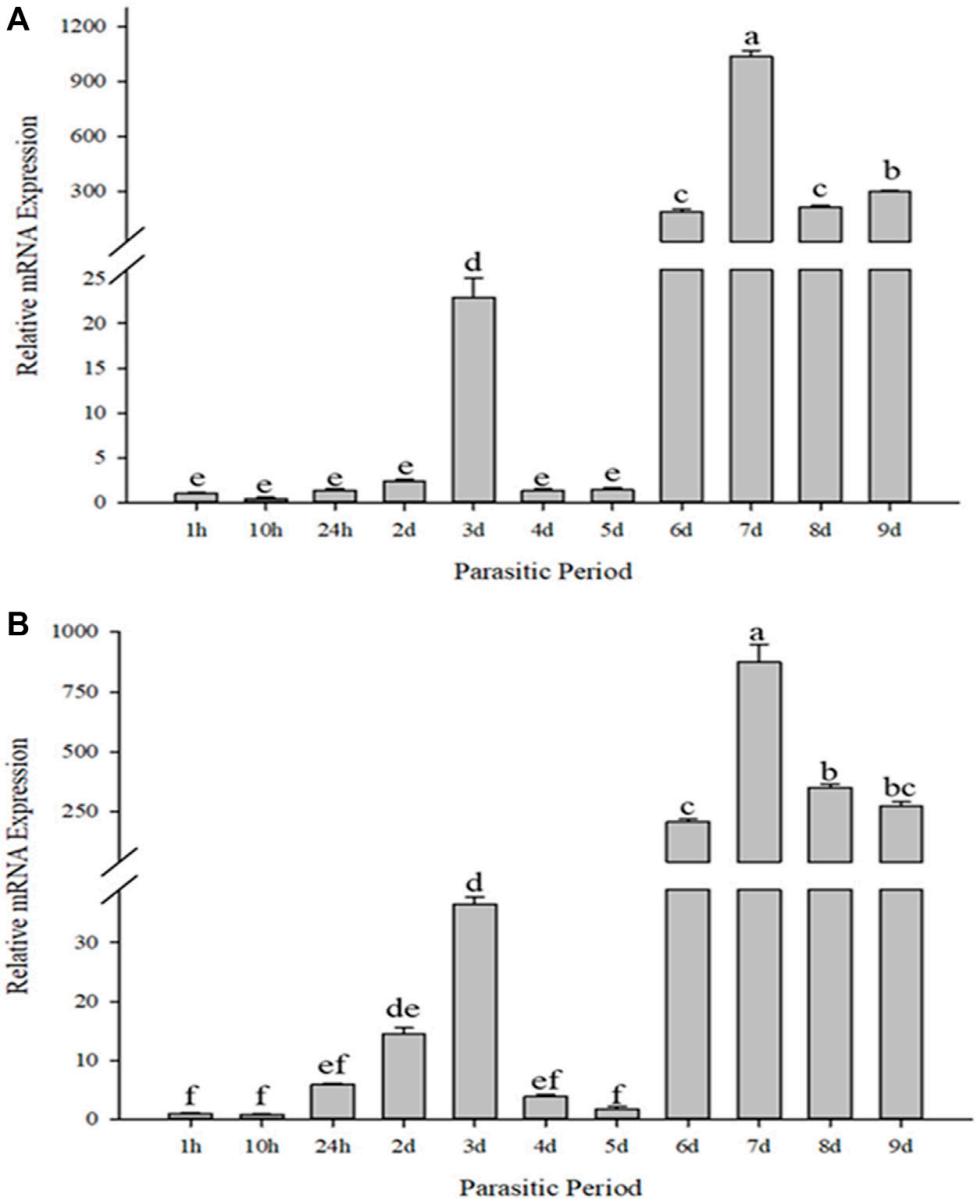
Relative mRNA expression levels of *Cc*IAP1 **(A)** and *Cc*IAP **(B)** in response to different parasitic time. Statistics represent means ± SE. Columns labeled with different letters indicate significant differences between times using one-way ANOVA followed by Tukey’s multiple comparison analysis (*p* < 0.05).

When compared for different durations of parasitism, *Cc*IAP1 and *Cc*IAP genes expression patterns were similar but not identical (Figure 4). When compared to the control (1 h of parasitism), *Cc*IAP1 gene was significantly upregulated at 3, 6, 7, 8 and 9 days of parasitism (*Cc*IAP1*: F*_10,33_ = 1869.450, *p* < 0.001); *Cc*IAP gene was significantly upregulated at 2, 3, 6, 7, 8 and 9 days (*Cc*IAP: *F*_10,33_ = 423.424, *p* < 0.001). The highest expression levels of *Cc*IAP1 and *Cc*IAP genes were observed at 7 days where levels were 1026.73- and 855.25-fold higher than the control (1 h), respectively.

### 3.5 Gene expression in response to different temperatures

**FIGURE 5:**
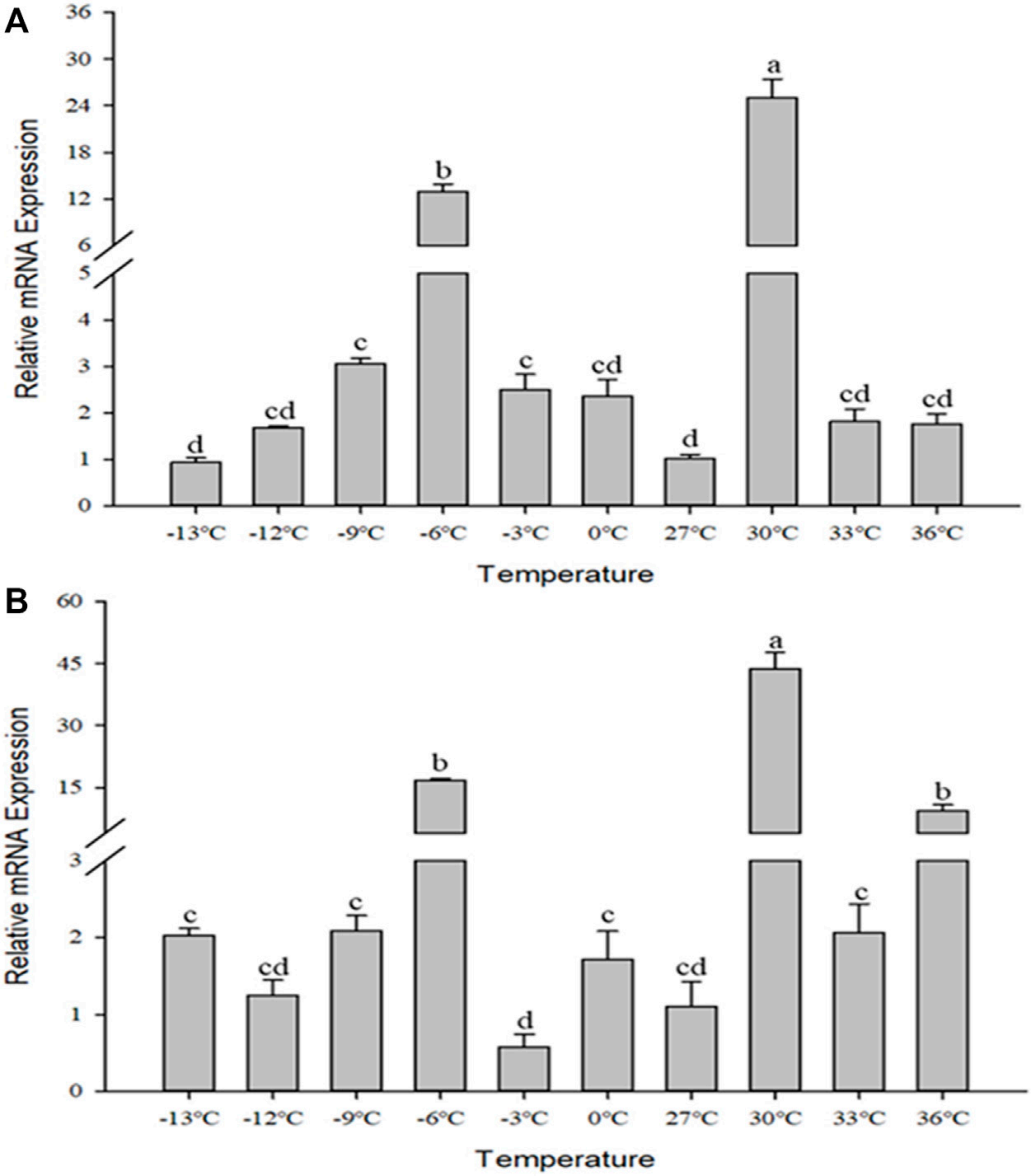
Relative mRNA expression levels of *Cc*IAP1 **(A)** and *Cc*IAP **(B)** at different temperatures. Data represent means ± SE. Columns labeled with different letters indicate significance using one-way ANOVA followed by Tukey’s multiple comparison analysis (*p* <0.05).

The relative mRNA levels of *Cc*IAP1 and *Cc*IAP genes were monitored at temperature gradients ranging from −13°C to 36°C. The expression of both genes were both upregulated by heat and cold stress (*Cc*IAP1: *F*_9,20_ = 132.603, *p* < 0.001; *Cc*IAP: *F*_9,20_ = 54.613, *p* < 0.001). *Cc*IAP1 gene was remarkably upregulated at −3°C, −6°C, −9°C, and 30°C (Figure 5A), and *Cc*IAP gene was significantly upregulated at −6°C and 30°C (Figure 5B). The expression levels of both *Cc*IAP1 and *Cc*IAP genes were highest at 30°C and were 24.81- and 39.56-fold greater than the control at 27°C, respectively.

### 3.6 Knockdown of *Cc*IAP1 and *Cc*IAP genes expression

**FIGURE 6:**
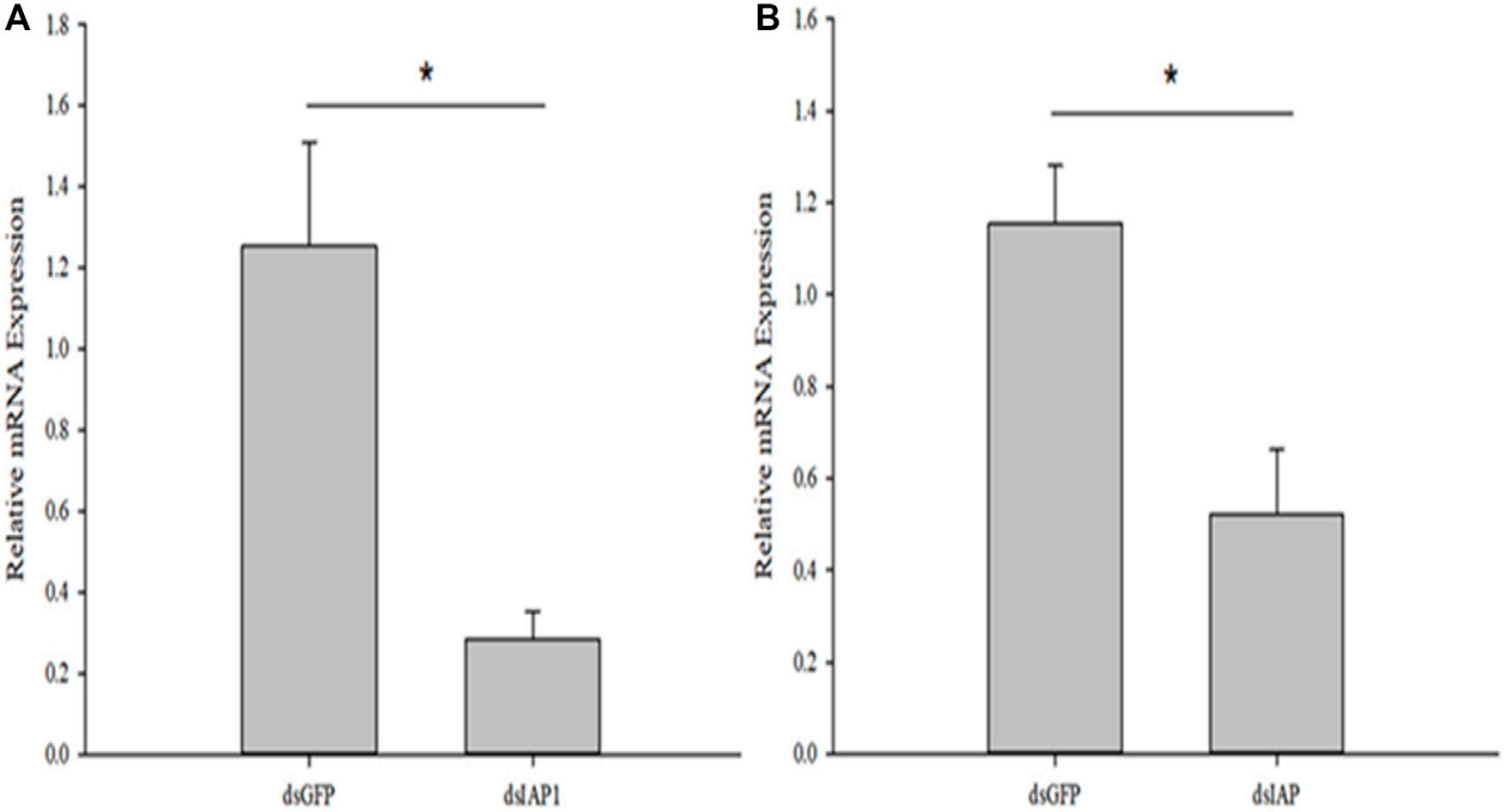
Relative mRNA expression levels of *Cc*IAP1 **(A)** and *Cc*IAP **(B)** in *Cotesia chilonis* after RNAi for 24 h. Statistics are presented as means ± SE. Data were analyzed using independent samples *t*-test built in SPSS software. *p* < 0.05 was considered statistically significant. Asterisks represent significant differences between control and RNAi treatment.

RNA interference studies were conducted by covering parasitized third instar larvae of *C. suppressalis* with a solution containing nanocarrier, dsRNA, and detergent, which successfully penetrated the body wall cavity of *C. suppressalis* (Supplementary Figure S1). The expression of *Cc*IAP1 and *Cc*IAP genes were both significantly reduced when the nanocarrier/ds*Cc*IAP1/detergent or nanocarrier/ds*Cc*IAP/detergent mixture was used for RNAi as compared to the nanomaterial/dsGFP/detergent control (*Cc*IAP1: *t* = 3.647, *p* < 0.05; *Cc*IAP: *t* = 3.357, *p* < 0.05) (Figure 6). However, the silencing efficiency of ds*Cc*IAP1 was higher than ds*Cc*IAP; treatment with the former reduced the *Cc*IAP1 transcript levels by 78% while the latter reduced *Cc*IAP levels by 55% as compared with the control.

**FIGURE 7:**
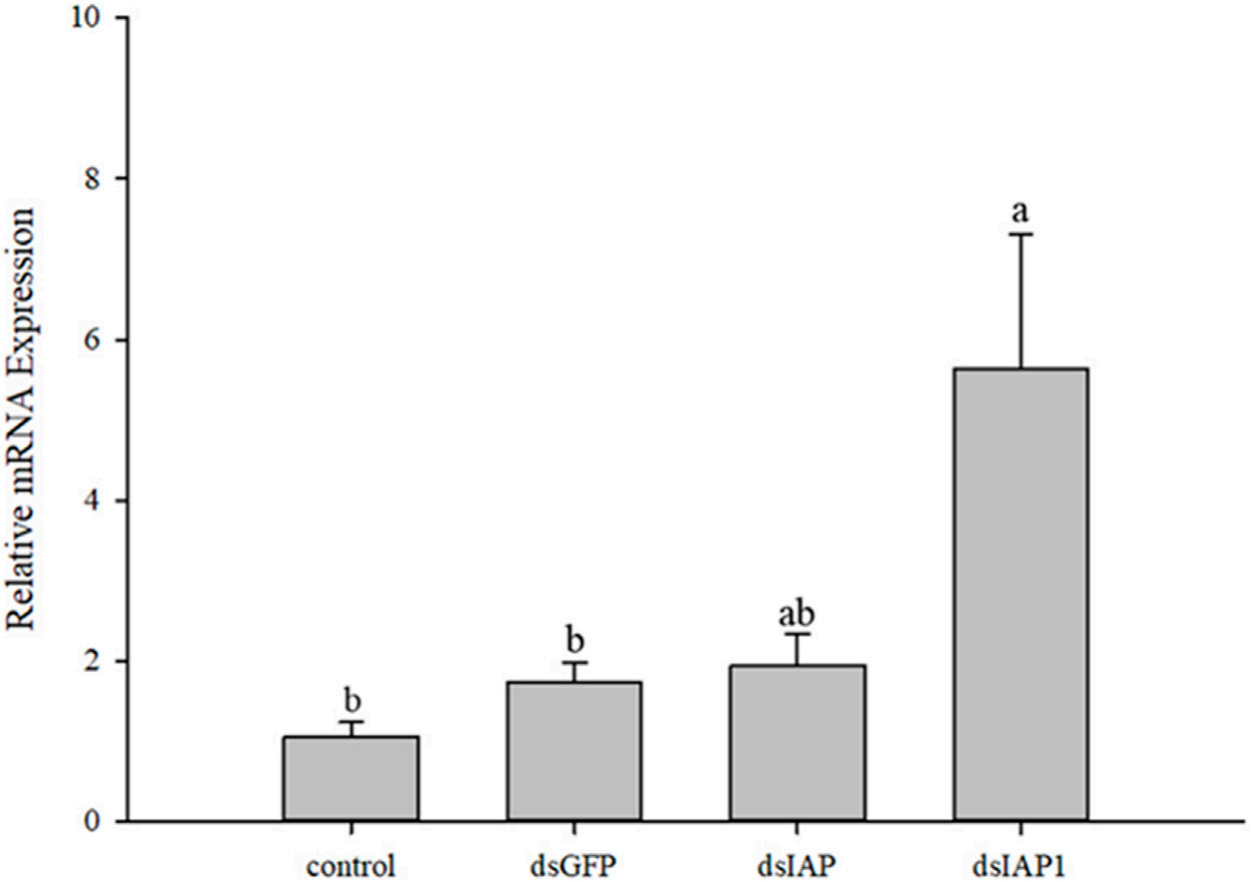
Relative mRNA expression levels of *Cscaspase-1* in *Chilo suppressalis* after silencing IAP genes in *Cotesia chilonis* for 24 h. Statistics represent means ± SE, and columns labeled with different letters indicate significant differences between different treatments using one-way ANOVA followed by Tukey’s multiple comparison analysis (*p* < 0.05).

There was a significant upregulation in expression of the apoptosis factor encoded by *Cscaspase-1* in parasitized *C. suppressalis* treated with the nanomaterial/ds*Cc*IAP1/detergent solution as compared to the control (*F*_3,12_ = 5.498, *p* < 0.05) (Figure 7). Interestingly, expression of the apoptosis effector encoded by *Cscaspase-1* was not significantly different from the dsGFP control when samples treated with the nanomaterial/ds*Cc*IAP/detergent mixture.

## 4 Discussion

Apoptosis is a genetically and biochemically controlled process and plays crucial roles in the development, issue homeostasis and defense of multicellular organisms. Inhibitor of apoptosis proteins (IAPs) are the class of proteins that can effectively inhibit cell apoptosis (Hamajima et al., 2016). In this study, we cloned and characterized a typical IAP1 gene (*Cc*IAP1) and an atypical IAP1 gene (*Cc*IAP). Multiple sequence alignments and phylogenetic analysis show that *Cc*IAP1 and *Cc*IAP are highly conserved and closely related to orthologues in other hymenopteran insects. *Cc*IAP1 contains two BIR domains and one RING domain while *Cc*IAP only contains two BIR domains, which are characteristic domains of IAPs that can bind one Zn^2+^, and its function is mainly to mediate the interaction between proteins. In addition, The BIR domains are divided into Type 1 and Type 2 according to whether there is a peptide binding groove. The Type 1 lacks the peptide binding groove and can interact with TRAF (tumor necrosis factor receptor-associated factor) and TGF-β (transforming growth factor-β) to regulate their signal pathways (Li et al., 2007). In this study, *Cc*IAP1 and *Cc*IAP both contain Type 2 BIR domains with unique hydrophobic clefts that can be combined with caspase or IAP binding motifs (IBMs) present in IAP antagonists (Verhagen et al., 2007). Most of RING domains that can bind two Zn^2+^ have E3 ubiquitin ligase activities (Feltham et al., 2011; Middleton et al., 2014; Takeda et al., 2014; Clem, 2015). Among them, the combination of BIR domain and caspase plus the participation of the RING domain with ubiquitin ligase (E3) activities can exert the full anti-apoptotic function of IAP (Schile et al., 2008). Furthermore, the two genes both lack introns in *C. chilonis*. And it has been reported that genes without introns or containing short introns may be expressed at higher levels than genes with multiple or long introns in response to stress (Comeron, 2004; Gao et al., 2019). Previous studies have shown that baculovirus IAP3, which has the most obvious anti-apoptosis function, has a close evolutionary relationship with insect IAPs (Birnbaum et al., 1994; Carpes et al., 2005; Liang et al., 2012). Our phylogenetic results suggest that baculovirus IAP3 genes might be derived from Lepidoptera, Diptera or Hymenoptera through horizontal gene transfer; this speculation is similar to previous results showing that Lepidoptera and Diptera are the primary hosts of baculoviruses (Chen et al., 2020).

The growing development and morphologic changes of organism are in closely connection with cell apoptosis (Elmore, 2007; Kerr et al., 2015). Apoptosis is a normal process in insect development, especially in holometabolous species, and plays a significant role in maintaining homeostasis. IAPs inhibit apoptosis via the BIR domains that mediate protein (Kerr et al., 2015). In different developmental stage experiments, the expression of *Cc*IAP1 and *Cc*IAP were both induced in the third instar larvae stage, which corresponded to predicted times of feeding and growth of *C. chilonis*. Moreover, *C. chilonis* that just parasitized have relatively sufficient nutrient support, but with the growth and development, their demand for living space and nutrients increases, and the competition among individuals intensifies. Therefore, it is reasonable to assume that the expression of *Cc*IAP1 and *Cc*IAP genes increased to meet nutritional demands in the third instar larval stage. Parasitism of *C. suppressalis* by *C. chilonis* caused a general increase in *Cc*IAP1 and *Cc*IAP genes expression, and mRNA levels at the 3, 6, 7, 8, and 9 days time points were significantly higher than the control, especially at the 7 days time point. Our results showed that the expression levels of *Cc*IAP1 and *Cc*IAP genes were higher in late larval stages as compared to earlier stages, which is consistent with the premise that *C. chilonis* may inhibit apoptosis in the late larval stages to foster growth and development. One explanation for elevated expression at the 3 days time point is to provide support for the egg-to-larvae transition. As for the downregulation at 4 and 5 days, we speculate that the needs of young *C. chilonis* larvae have little effect on the development of *C. suppressalis* and the host-parasite interaction is somewhat balanced at these time points. In organisms, IAPs bind to active caspases that execute the cell death program, and prior research has shown that caspases function in regulation of the mitochondrial apoptotic pathway (Ehrmann et al., 2023). In this study, *Cc*IAP1 and *Cc*IAP genes were both silenced by nanocarrier and ds*Cc*IAP1 or ds*Cc*IAP. These results indicated that nanocarrier could help dsRNA pass through the host silence the objective genes of wasp in the host. Further analyses exhibited *Cscaspase-1* expression in *C. suppressalis* was significantly upregulated when *Cc*IAP1 gene was silenced, which suggests that *Cc*IAP1 could contribute to the inhibition of apoptosis in *C. suppressalis*. In a related study, the induction of apoptosis could be blocked by the overexpression of *Sf-*IAP1 in *Spodoptera frugiperda* (Veeran et al., 2020). Furthermore, our results show that *Cscaspase-1* and *Cc*IAP1 play a vital role in the parasitoid-pest interaction, and these complex interactions warrant further study.

Temperature is a critical environmental factor that affects insect growth, development, distribution and abundance (Bale et al., 2002). When subjected to high or low temperature stress, insects may adopt different coping strategies, such as avoidance behaviors or changing physiological functions to tolerate temperature stress (Hoffmann and Parsons, 1991). Some studies shown that external stimulus signals could mediate cell apoptosis, and XIAP play a synergistic role in the mechanism of pro-apoptosis (Igaki et al., 2002; Ludivine et al., 2010). And IAPs are at the center of this system to preserve sudden damages and death (Hanifeh and Ataei, 2022). In this study, we found that the expression of *Cc*IAP1 and *Cc*IAP genes were both significantly upregulated at −6°C and 30°C in response to temperature stress, well indicating that temperature as the abiotic stress plays a part in the apoptosis pathway of cells and the temperature tolerance is also related to the expression and regulation of IAP genes in insects, which had not been shown before. Our experimental results also indicated that temperature tolerance may have evolved in response to long exposure to moderately high or low temperatures rather than to an acute bout of extremely high or low temperatures, which is similar to the results of previous studies (Addo-Bediako et al., 2000; Hance et al., 2007). In addition, heat shock proteins have been reported to regulate apoptosis and cell death (Takayama et al., 2003), suggesting that the mechanism of temperature stress between heat shock proteins and inhibitor of apoptosis proteins is a great direction worthy of study.

In conclusion, two IAP genes (*Cc*IAP1 and *Cc*IAP) in *C. chilonis* were firstly cloned and characterized, both of them were expressed during different developmental stages, especially in the third instar larvae stage. And the expression of them were both significantly upregulated in response to temperature stress and parasitism. The current study furthers our understanding of the regulation of IAP genes of insect under development and temperature stress, and provides groundwork for future studies aimed at understanding the molecular mechanisms that are driving the evolutionary adaptation of *C. chilonis*.

## Data Availability

The original contributions presented in the study are included in the article/Supplementary Material, further inquiries can be directed to the corresponding author.
