# Supplementary material for: The regulation of inhibitor of apoptosis proteins (IAPs) during the apoptosis of Cotesia chilonis
Source: Front Physiol. 2023 Dec 19;14:1328167. doi: 10.3389/fphys.2023.1328167 (PMC10773855; doi:10.3389/fphys.2023.1328167)
Supplement: Supplementary file 1 [file DataSheet3.docx]

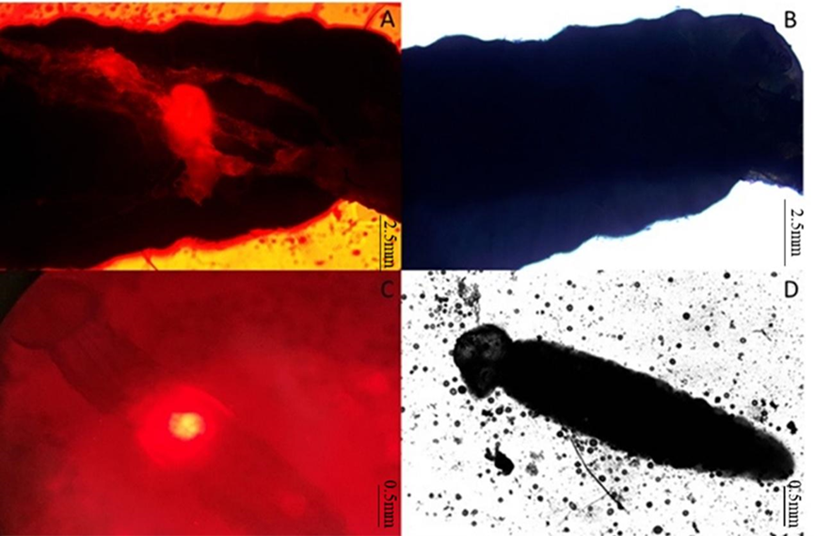


**Figure S1: Nanomaterial/dsRNA complex penetrated body wall into cavity.**

(**A**) The red fluorescence of *Chilo suppressalis* larvae coelom under fluorescence; (**B**) The image of *Chilo suppressalis* larvae coelom without fluorescence; (**C**) The red fluorescence of *Cotesia chilonis* larvae under fluorescence; (**D**) The image of *Cotesia chilonis* larvae without fluorescence.
