# Supplementary material for: The regulation of inhibitor of apoptosis proteins (IAPs) during the apoptosis of Cotesia chilonis
Source: Front Physiol. 2023 Dec 19;14:1328167. doi: 10.3389/fphys.2023.1328167 (PMC10773855; doi:10.3389/fphys.2023.1328167)
Supplement: Supplementary file 2 [file DataSheet2.docx]

**Table. S2** Details of species compared in this study.

| **Species** | **Protein Description** | **Accession No.** | **Protein (aa)** |
| --- | --- | --- | --- |
| **Baculovirus IAP3** |  |  |  |
| *Adoxophyes orana* granulovirus | AdorGV *iap3* | NP_872543.1 | 254 |
| *Agrotis segetum* nucleopolyhedrovirus | AgseNPV *iap3* | YP_529789.1 | 271 |
| *Anticarsia gemmatalis* nucleopolyhedrovirus | AngeNPV *iap3* | YP_803428.1 | 287 |
| *Buzura suppressaria* nucleopolyhedrovirus | BusuNPV *iap3* | YP_009001870.1 | 276 |
| *Chrysodeixis chalcites* nucleopolyhedrovirus | ChchNPV *iap3* | YP_249643.1 | 278 |
| *Cryptophlebia leucotreta* granulovirus | CrleGV *iap3* | NP_891863.1 | 255 |
| *Epinotia aporema* granulovirus | EpapGV *iap3* | YP_006908519.1 | 256 |
| *Epiphyas postvittana* nucleopolyhedrovirus | EppoNPV *iap3* | NP_203195.1 | 261 |
| *Erinnyis ello* granulovirus | ErelGV *iap3* | YP_009091936.1 | 269 |
| *Hyphantria cunea* nucleopolyhedrovirus | HycuNPV *iap3* | YP_473308.1 | 263 |
| *Leucania separata* nucleopolyhedrovirus | LeseNPV *iap3* | YP_758337.1 | 248 |
| *Pseudoplusia includens* SNPV IE | PsinSNPV *iap3* | YP_009116952.1 | 282 |
| *Spodoptera frugiperda* MNPV | SpfrMNPV *iap3* | YP_001036403.1 | 287 |
| **Lepidoptera** |  |  |  |
| *Bombyx mori* | *iap2* | XP_012551311.1 | 585 |
| *Chilo suppressalis* | *iap1* | unpublished | 566 |
| *Lymantria dispar* | *iap1* | BAM63312.1 | 362 |
| *Manduca sexta* | *iap1* | XP_030025462.2 | 373 |
| *Mythimna separata* | *iap1* | BAM76810.1 | 379 |
| *Spodoptera exigua* | *iap1* | ABA62322.1 | 378 |
| *Spodoptera litura* | *iap1* | AFA43941.1 | 378 |
| *Trichoplusia ni* | *iap1* | AAF19819.1 | 379 |
| **Diptera** |  |  |  |
| *Anopheles gambiae* | *iap1* | XP_308527.5 | 369 |
| *Culex pipiens* | *iap1* | ABP35673.1 | 409 |
| *Glossina morsitans morsitans* | *iap2* | ABC25071.1 | 526 |
| **Hymenoptera** |  |  |  |
| *Apis cerana* | *iap2* | *XP_016921505.2* | 824 |
| *Apis mellifera* | *iap2* | XP_396819.2 | 518 |
| *Bombus impatiens* | *iap1* | XP_003492055.1 | 406 |
| *Bombus terrestris* | *iap2* | XP_020718439.1 | 546 |
| *Bombus terrestris* | *iap1* | XP_003393061.1 | 406 |
| *Dufourea novaeangliae* | *iap1* | XP_015432903.1 | 407 |
| *Dufourea novaeangliae* | *iap2* | KZC11010.1 | 549 |
| *Habropoda laboriosa* | *iap1* | KOC68082.1 | 401 |
| *Habropoda laboriosa* | *iap2* | KOC68081.1 | 543 |
| *Melipona quadrifasciata* | *iap1* | KOX74162.1 | 398 |
| *Melipona quadrifasciata* | *iap2* | KOX74161.1 | 524 |
| *Nasonia vitripennis* | *iap1* | XP_031782270.1 | 359 |
