# Supplementary material for: The regulation of inhibitor of apoptosis proteins (IAPs) during the apoptosis of Cotesia chilonis
Source: Front Physiol. 2023 Dec 19;14:1328167. doi: 10.3389/fphys.2023.1328167 (PMC10773855; doi:10.3389/fphys.2023.1328167)
Supplement: Supplementary file 3 [file DataSheet1.docx]

**Table S1** Primers used in this study.

| Primer name | Primer sequences (5’-3’) |
| --- | --- |
| Fragment verification |  |
| IAP1-F | TACGAGAGTGGGTAGAGGGTGA |
| IAP1-R | ACGGGCTCTCTACACACTGGGC |
| IAP-F | TTCAGCCAGCGTCGCATTTAC |
| IAP-R | AGTCATACTTGCCGCACACCT |
| IAP1-5’ | CCTTTAGGCTGGGCGAGGTTTT |
| IAP1-3’ | AAGTCGGTCAACAAGAGTGCGGA |
| IAP-5’ | CAGTCTCACAGATTCCAGGCGGTA |
| IAP-3’ | AAACTGTCTGCTATCACTGTGGCGG |
| IAP1-F | ACGCAGAGTACATGGGGAGT |
| IAP1-R | TGTAAAGGTACTAAGATATTATAGCACG |
| IAP-F | TGCAGCATCATTTTCATCTTCA |
| IAP-R | TTCTTCTCCTCGGGTTTCTTT |
| Verification of genome |  |
| IAP1-F | TGACGCACATAACTGCTGTT |
| IAP1-R | TTTTTTGATGTAAAGGTACTAAGA |
| IAP-F | TGCAGCATCATTTTCATCTTCA |
| IAP-R | TTCTTCTCCTCGGGTTTCTTT |
|  |  |
| Real-time quantitative PCR |  |
| IAP1-F | CGAGAGTGGGTAGAGGGTGA |
| IAP1-R | TCAATCGGCACGTTACCACA |
| IAP-F | GGCAGTCCAATTGCCGTTTT |
| IAP-R | ACGTCACTGCCTCTAGGACT |
| Caspase-1-F  Caspase-1-R | TGCATGTCATTTTCGGTTGTTG  CTCCGCCGTTTTCCCTTTG |
| Reference gene in qRT-PCR |  |
| H3-F | CGTCGCTCTTCGTGAAATCA |
| H3-R | TCTGGAAACGCAAGTCGGTC |
| GAPDH-F | GAAGGTGGTGCCAAGAAAG |
| GAPDH-R | GCATGGACAGTGGTCATAAGA |
| dsRNA synthesis  dsIAP1-F  dsIAP1-R  dsIAP-F  dsIAP-R  dsGFP-F  dsGFP-R | TAATACGACTCACTATAGGG  CACATAACTGCTGTTTTTGTCG  TAATACGACTCACTATAGGG  CAGATTTGCCGTTGGCTTTA  TAATACGACTCACTATAGGG  CATAACTGCTGTTTTTGTCGA  TAATACGACTCACTATAGGG  ATTGTTGGGTAAATGCGACG  TAATACGACTCACTATAGGG  CCTCGTGACCACCCTGACCTAC  TAATACGACTCACTATAGGG  CACCTTGATGCCGTTCTTCTGC |

Note: The qPCR primers used in this study were validated (Li et al., 2019), the underline indicates the T7 promoter sequence.
